# Thermal Hazard Evaluation of Cumene Hydroperoxide-Metal Ion Mixture Using DSC, TAM III, and GC/MS

**DOI:** 10.3390/molecules21050562

**Published:** 2016-04-28

**Authors:** Mei-Li You

**Affiliations:** Department of General Education Center, Chienkuo Technology University, 1 Chieh-Shou N. Rd., Changhua 50094, Taiwan; mei@ctu.edu.tw

**Keywords:** cumene hydroperoxide (CHP), organic peroxide, differential scanning calorimetry (DSC), thermal activity monitor III (TAM III), gas chromatography/mass spectrometer (GC/MS)

## Abstract

Cumene hydroperoxide (CHP) is widely used in chemical processes, mainly as an initiator for the polymerization of acrylonitrile–butadiene–styrene. It is a typical organic peroxide and an explosive substance. It is susceptible to thermal decomposition and is readily affected by contamination; moreover, it has high thermal sensitivity. The reactor tank, transit storage vessel, and pipeline used for manufacturing and transporting this substance are made of metal. Metal containers used in chemical processes can be damaged through aging, wear, erosion, and corrosion; furthermore, the containers might release metal ions. In a metal pipeline, CHP may cause incompatibility reactions because of catalyzed exothermic reactions. This paper discusses and elucidates the potential thermal hazard of a mixture of CHP and an incompatible material’s metal ions. Differential scanning calorimetry (DSC) and thermal activity monitor III (TAM III) were employed to preliminarily explore and narrate the thermal hazard at the constant temperature environment. The substance was diluted and analyzed by using a gas chromatography spectrometer (GC) and gas chromatography/mass spectrometer (GC/MS) to determine the effect of thermal cracking and metal ions of CHP. The thermokinetic parameter values obtained from the experiments are discussed; the results can be used for designing an inherently safer process. As a result, the paper finds that the most hazards are in the reaction of CHP with Fe^2+^. When the metal release is exothermic in advance, the system temperature increases, even leading to uncontrollable levels, and the process may slip out of control.

## 1. Introduction

Since the 1970s, the chemical industry has been booming in Taiwan, and it has contributed to the industrial development and economic progress of the country. However, we sporadically hear of disastrous accidents in the chemical process industry [1,2,3]. In fact, any manufacturing process potentially possesses a variety of risks. Therefore, minimizing risks and creating a safer, worry-free, friendly working environment was the main objective of this study and the ultimate goal.

**Figure 1:**
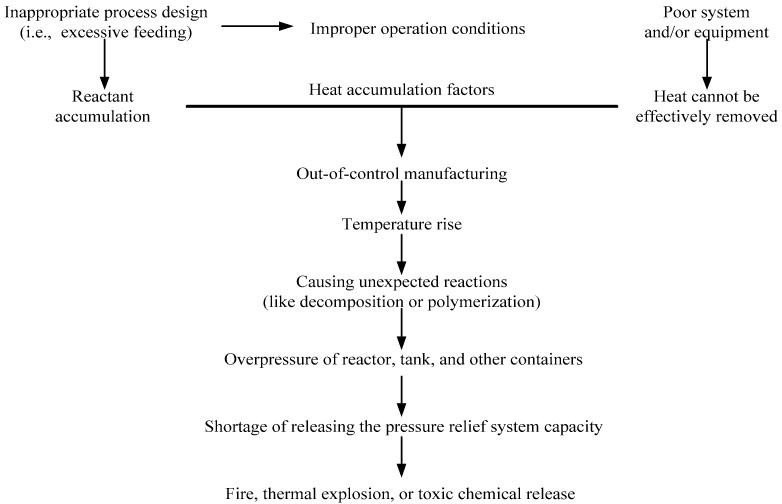
Illustration of a process leading to an out-of-control reaction.

In practice, cumene hydroperoxide (CHP) is an initiator for the polymerization of acrylonitrile–butadiene–styrene (ABS), which has wide applications and is ubiquitous in our daily life. CHP is organic peroxide for the following reasons: its structure has a peroxy bond (–O–O–) [4], it has high thermal sensitivity, and it is susceptible to the catalytic properties of contaminants [5,6,7,8]. During the manufacturing process, if the process is aging or other uncontrollable factors such as CHP with incompatible chemicals during their exposure to the substance, it may therefore cause unexpected exothermic reaction in advance [9,10]. Out-of-control reaction processes are normally a result of improper control of the manufacturing process, or problems in operating equipment, and, therefore, heat is accumulated in the system. Figure 1 illustrates a process leading to an out-of-control reaction. 

In the past, manufacturing processes used for such substances have repeatedly caused casualties or equipment damage. Table 1 lists relevant disaster events that have occurred worldwide since 2001.

An organic-peroxide-based structure has an unstable double bonding group that can readily decompose with exothermic characteristics. CHP itself is incompatible, which makes it react with acids, bases, metal ions, or other substances [9,10,11].

Corrosion results from the surface of metal undergoing a chemical or electrochemical reaction with the substances present in the environment, which leads to the gradual erosion of the metal interior [12,13]. Corrosion releases metallic materials [14,15], and it can be considered to be equivalent to adding a solution of metal ions. According to the literature, the commonly used materials for fabricating metal pipes are carbon steel (carbon ferrochrome and manganese), steel, chrome, stainless steel, and tin (galvanized iron stainless steel). In the past, there have been several cases of damage caused by plague display pipeline corrosion. On 10 July 2011, several fires occurred at a petrochemical industrial complex along the west coast in Taiwan because the corrosion of the central coastal pipeline. Subsequently, the authorities ordered the replacement of all pipelines. The pipes at the plant were changed to hot-dip galvanized steel pipes. On 30 July 2014, the shocking Kaohsiung propylene gas explosion disaster occurred because the underground pipeline had been corroded by water and gas over several decades. The corrosion caused the pipeline to rupture, leading to the leakage of enormous amount of propylene. The leakage left 32 people dead and 321 injured. The line connecting often requires welding, and substances such as iron, nickel, zinc, copper, manganese, zinc, lead, and chromium are used for filling.

The main objective was to conduct dynamic research to study the stability of the CHP with metal ions, that generated by corrosion phenomena. 

## 2. Results and Discussion

### 2.1. Thermal Analysis by DSC

**Figure 2:**
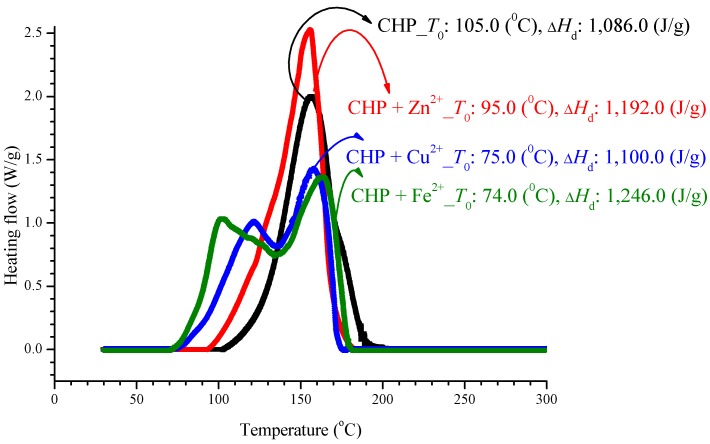
Heat flow *versus* temperature plot for 80.0 mass% CHP mixed with ZnBr_2_, CuBr_2_, or FeBr_2_, tested using differential scanning calorimetry (DSC) analysis at a heating rate of 4.0 °C/min.

In an experiment, CHP was added to three incompatible metals, and the aforementioned method was used to achieve thermal decomposition, as shown in Figure 2. We can find advanced phenomena on *T*_0_ for mixed metal ions of sample. However, there is no appreciable difference in Δ*H*_d_ and *T*_max_. After adding CHP to ZnBr_2_, CuBr_2_, or FeBr_2_, *T*_0_ increased to 95.0, 75.0, and 74.0 °C, respectively. For these additions to ZnBr_2_, CuBr_2_, and FeBr_2_, the Δ*H*_d_ values were 1192.0, 1100.0, and 1246.0 J/g, respectively. The parameter *T*_max_ was stable at 160.0 ± 5.0 °C, and the relevant values are listed in Table 2.

After adding metal ions to CHP, it will let the exothermic onset temperature (*T*_0_), in advance of appeared from 74.0–95.0 °C, as shown in Table 2. The exothermic onset temperature (*T*_0_) of CHP is 105.0 °C. Hence, metal ions can be said to cause the reaction that affects CHP, let it *T*_0_ was in advance. It is noteworthy that, apart from the ion curves of Cu^2+^ and Fe^2+^, *T*_0_ can have advanced phenomenon, and its peak value is far from the single peak. However, Δ*H*_d_ is maintained within 1150.0% ± 10% J/g, where 10% is the experimental error. The reason is that, in this test, only CHP is used to provide heat, and the metal ion does not generate heat, and therefore, the metal ion does not affect their heat of decomposition.

During the production process, CHP and the mixed metal ions are scrambled, and this may cause an advanced reaction. A substance will have out of control phenomenon earlier than the preset temperature. The second peak of Cu^2+^ and Fe^2+^ may also involve detrimental effects. If the protection system fails to monitor the production process, it will make the process go out of control, resulting in a major disaster.

### 2.2. Isothermal Hazard Analysis by TAM III

**Figure 3:**
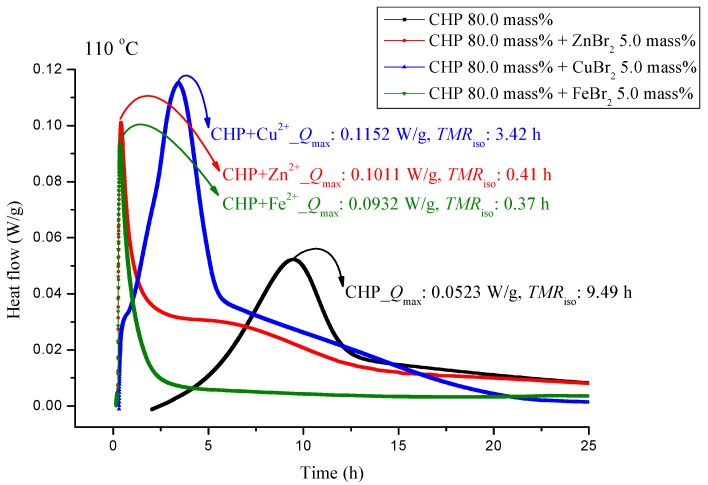
Heat flow *versus* temperature plot for 80.0 mass% CHP mixed with ZnBr_2_, CuBr_2_, or FeBr_2_, tested using TAM III scanning analysis at 110.0 °C.

**Figure 4:**
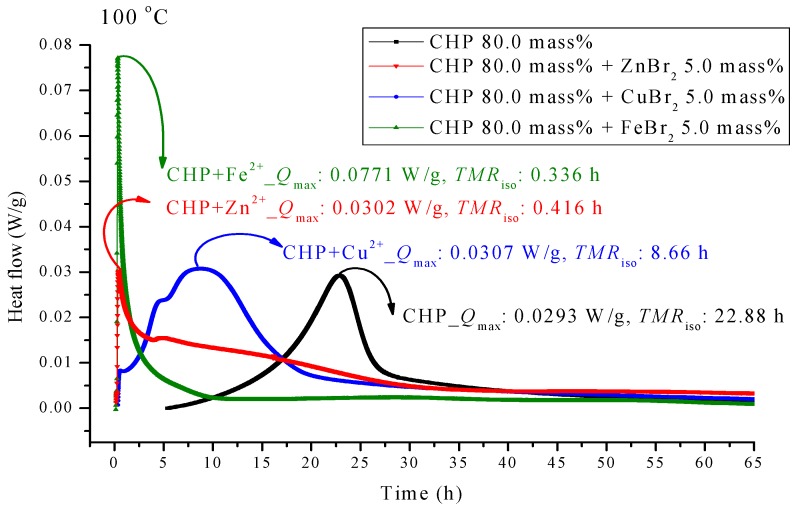
Heat flow *versus* temperature plot for 80.0 mass% CHP mixed with ZnBr_2_, CuBr_2_ or FeBr_2_, tested using TAM III scanning analysis at 100.0 °C.

**Figure 5:**
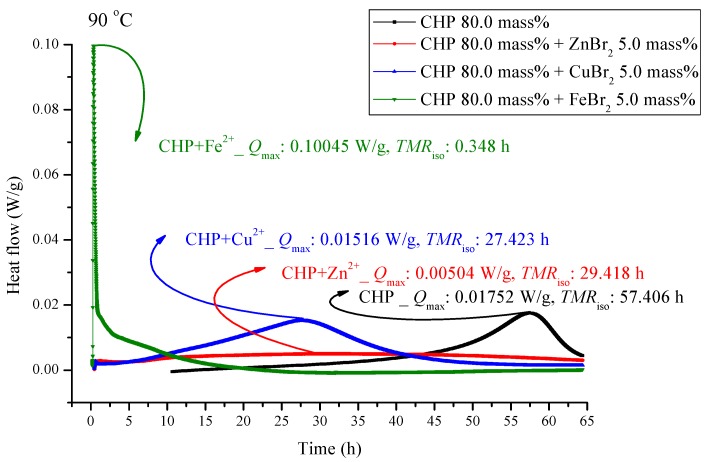
Heat flow *versus* temperature plot for 80.0 mass% CHP mixed with ZnBr_2_, CuBr_2_, or FeBr_2_, tested using TAM III scanning analysis at 90.0 °C.

**Figure 6:**
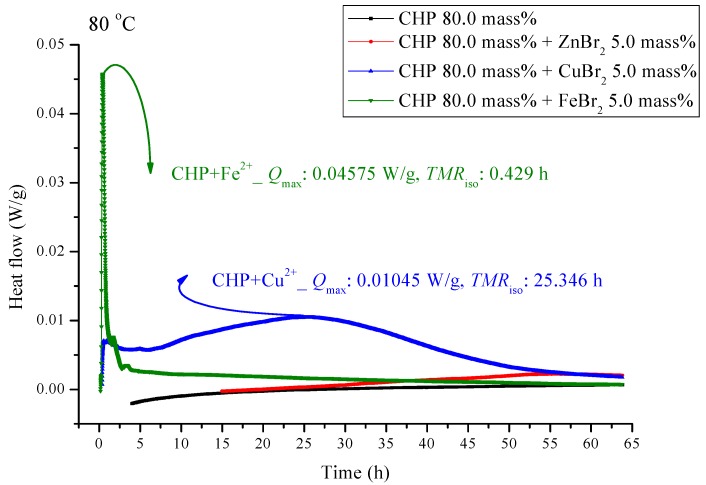
Heat flow *versus* temperature plot for 80.0 mass% CHP mixed with ZnBr_2_, CuBr_2_, or FeBr_2_, tested using TAM III scanning analysis at 80.0 °C.

The experiments involved the pure substance 80.0 mass% CHP, which was maintained under 110.0 °C in a constant temperature environment in TAM III. TAM III was used to investigate the runaway reaction of CHP mixed with ZnBr_2_, CuBr_2_, or FeBr_2_ by recording the associated heat generated under isothermal conditions at temperatures of 80.0, 90.0, 100.0 and, 110.0 °C (Figure 3, Figure 4, Figure 5 and Figure 6). In Figure 3, the CHP thermostatic peak value can be seen to be 0.0523 W/g, which corresponds to 9.49 h. Metal ions were then added to CHP. In Figure 3, *Q*_max_ clearly increases to 0.1152 W/g and *TMR*_iso_ is approximately 3.42 h. The parameter *TMR*_iso_ is 0.41 and 0.37 h at the *Q*_max_ values of 0.1011 and 0.932 W/g, respectively. From Figure 3, we found that metal ions were added to CHP. The exothermic onset temperature occurred earlier, and the heat release was greater than twice that for pure CHP.

Figure 4 presents the TAM III pattern parallel to CHP with metal ions; the pattern was analyzed at 100.0 °C. We found that the metal ions forced *TMR*_iso_ to occur earlier. Among the metal ions, those of FeBr_2_ can be observed on the leftmost side. *TMR*_iso_ was 0.34 h, which was earlier than that of pure CHP by a factor of 0.68. ZnBr_2_ also showed a similar phenomenon. Its *TMR*_iso_ was 0.42 h, almost coinciding with the time of heat release. The *TMR*_iso_ value of CuBr_2_ was 8.66 h, which was earlier than that of pure CHP by a factor of only 2.6.

Figure 5 and Figure 6 are shown TAM III thermostat tests at 90.0 and 80.0 °C, respectively. The peak of CHP mixed with FeBr_2_ is tilted to the right, and when pure CHP is mixed with ferrous ions, the peak is the same as that at temperatures less than 100.0 and 110.0 °C. Because the heat value is considerably higher than that for other materials, it is dangerous and detrimental at temperatures less than 90.0 °C. The heat release for CHP mixed with CuBr_2_ and for pure CHP was approximately 0.016 ± 0.001 W/g. However, the peak of zinc ions is not very obvious, amounting to only 0.00504 W/g. Ferrous ions are added, it can reach 0.10045 W/g, and the heat release increases appreciably. When relevant disasters occur, the extent of harm may increase sharply. Figure 6 shows a thermostat experiment conducted at a temperature below 80.0 °C. For this condition, only peaks of iron and zinc ions remain. There is no heat release for pure CHP and the addition of zinc ions. We can predict that in the thermostat case at 80.0 °C, no reaction will occur.

CHP was used in the isothermal evaluation by using a TAM III scanning analysis at 80.0, 90.0, 100.0, and 110.0 °C. Table 3 presents thermostat experiments involving the addition of three metal ions. For example, at 110.0 °C, *Q*_max_ of CHP is only 0.0523 W/g. After the addition of metal ions, *Q*_max_ shows an approximately two–fold increase at *Q*_max_. The time to maximum rate at isothermal conditions (TMR_iso_) for the scanning analysis at 90.0, 100.0, and 110.0 °C were 57.41, 22.88, and 9.49 h. The TMR_iso_ values for cumene hydroperoxide (CHP) mixed with FeBr_2_, tested using TAM III scanning analysis at 80.0, 90.0, 100.0, and 110.0 °C were 0.34–0.43 h. Metal ions affect the reactions involving CHP by increasing the reactivity of CHP, and they change the heat mechanism markedly—especially, CHP added with incompatibility material FeBr_2_. The process should be performed very carefully once the system in the incompatible sections is operated in such a real hazard. CHP is affected by the addition of metals. Storage tanks, pipelines, and reactors used in the manufacture of CHP are all made of metals, and contact of the metals with CHP is inevitable.

### 2.3. Calculation of Thermokinetic Parameters

The heat determined in a reaction kinetics analysis of substances can be used to enhance the safety of the process design. Each stage of the process may be simulated by assuming faulty conditions, and the simulation results can be used for improving the processing phase, storage, and transport. In these experiments, the TAM III thermostat obtained the relevant parameters through calculations involving the Arrhenius equation and the apparent activation energy.

In the thermostat tests performed in this study, 80.0 mass% CHP was considered at 80.0, 90.0, 100.0, 110.0 °C and the following equation was used to calculate the apparent activation energy (*E_a_*):

(1) $$k_{0}=A\times \exp(\frac{-E_{a}}{RT})$$

where *k*_0_ is the reaction rate constant, *A* is the frequency factor, *E_a_* is the apparent activation energy, *R* is the ideal gas constant, and *T* is the absolute temperature.

Taking the natural logarithm on both sides of Equation (1) gives:

(2) $$\ln k_{0}=\ln A-(\frac{E_{a}}{RT})$$

In the Arrhenius equation, *E_a_* and *A* are the only constant values that do not change with the temperature. The greater the temperature, the reactions are faster. Therefore, the following equation can be regarded as a set of dynamic equations:

(3) $$\ln k_{1}+(\frac{E_{a}}{RT_{1}})=\ln k_{2}+(\frac{E_{a}}{RT_{2}})=\ln k_{3}+(\frac{E_{a}}{RT_{3}})=\cdots$$

where *T*_1_, *T*_2_, *T*_3_ ... are the temperatures in the absolute experiment and *k*_1_, *k*_2_, *k*_3_ ... are constants at the temperatures *T*_1_, *T*_2_, *T*_3_ ..., respectively.

From the results obtained using the Arrhenius equation and apparent activation energy of the experimental group, we can derive the results, as displayed in Table 4. The *E_a_* value of pure CHP is 155.5 kJ/mol. Furthermore, adding a metal layer containing materials incompatible with CHP leads to a decrease in *E_a_*, and the order of *E_a_* values was ZnBr_2_ (146.8 kJ/mol), CuBr_2_ (95.6 kJ/mol), and FeBr_2_ (21.8 kJ/mol). The minimum energy required to kick off a chemical reaction is called apparent activation energy. When molecules collide, the kinetic energy of the molecules can be used to bend, stretch, and ultimately break bonds, leading to chemical reactions. The lowering of apparent activation energy of CHP with Fe^2+^ means that they are easy to start to react compared with the other ions.

### 2.4. Literature Comparison and Verification

To verify the accuracy of this experiment, Table 5 displays a comparison of *E_a_* of CHP, obtained in the present study, with the values obtained from the previous studies.

### 2.5. Pyrolysis Products According to CHP Analysis by GC/MS

Organic peroxides are widely used in industrial processes because of their high reactivity. Frequent disasters related to the manufacture of organic peroxides may also occur. CHP is prevailingly used for the manufacture of phenol, acetone, α-methylstyrene (AMS), and other common chemicals. To understand the corrosion of metal containers and release of metal ions during manufacturing process, we chose the trace amount of Zn^2+^, Cu^2+^, Fe^2+^ as experimental factors, and finally evaluated thermal cracking products by GC/MS analysis to determine product profiles.

**Figure 7:**
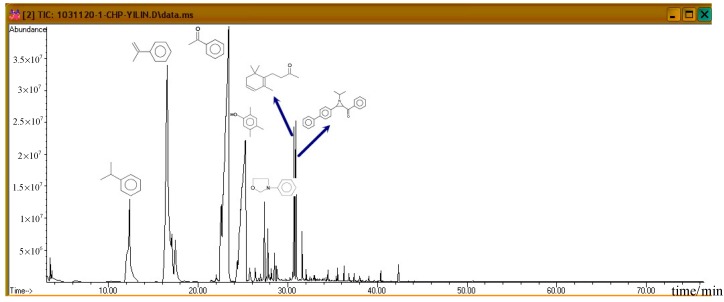
Product analysis of CHP decomposition using GC/MS.

Figure 7 shows the ion spectra of diluted 80.0 mass% CHP, after the completion of GC/MS analysis. For the default analysis and within 35.5 min, seven distinct crests can be observed. First, at 12.243 min, the first peak is cumene. Then, at 16.564 min, AMS is isolated, and it is followed by the third product acetophenone (AP) at 23.337 min. These three products are repeated in a subsequent atlas, and all of them are common derivatives or byproducts during the CHP manufacturing process. At 25.274 min, we found that phenol, 2,4,5-trimethyl, appeared, and at 27.383 min, oxazolidine, 3–phenyl, was formed. 

**Figure 8:**
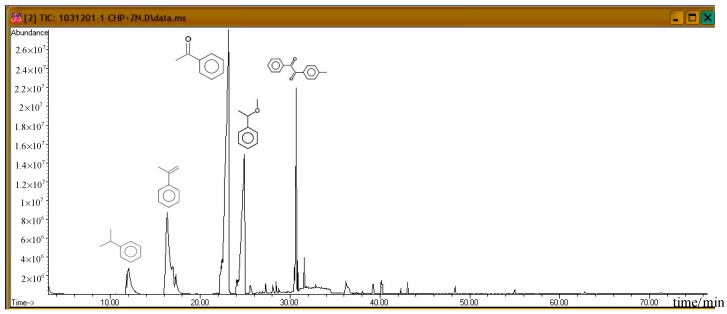
Product analysis of CHP decomposition after mixing CHP with ZnBr_2_ by GC/MS.

**Figure 9:**
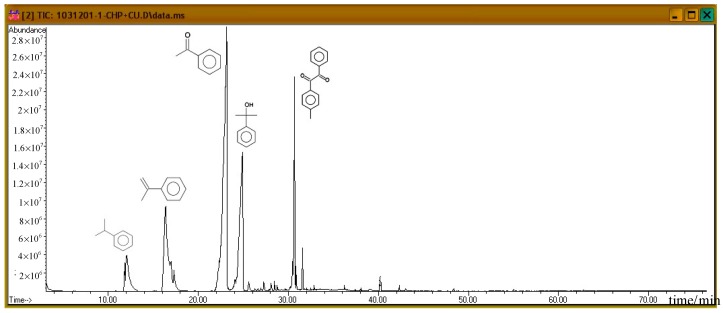
Product analysis of CHP decomposition after mixing CHP with CuBr_2_ by GC/MS.

**Figure 10:**
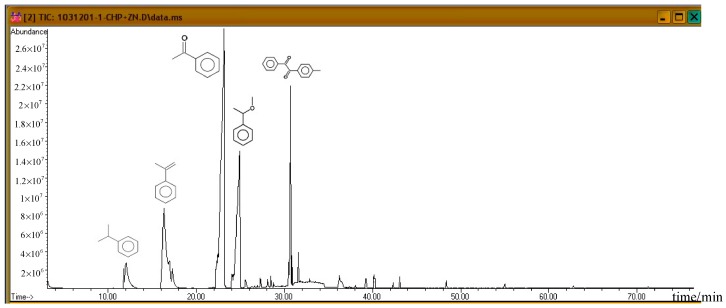
Product analysis of CHP decomposition after mixing CHP with FeBr_2_ by GC/MS.

Subsequently, the three incompatible metal materials were added. Figure 8, Figure 9 and Figure 10 delineate the spectra of diluted CHP with ZnBr_2_, CuBr_2_, and FeBr_2_, obtained through GC/MS analysis. Like the aforementioned atlas of pure substances, at 12.243, 16.564, and 23.337 min, the three substances—cumene, AMS, and AP—appear sequentially. Cumene is presumed to be a product of thermal volatilization. In addition, the spectrum obtained for the addition of zinc ions at 24.914 min is that of phenylethyl methyl ether. Furthermore, at 30.670 min, ethanedione, (4-methylphenyl) phenyl, appears. The spectrum of copper ions, α-cumyl alcohol (CA), appears at 24.914 min, and ethanedione, (4-methylphenyl) phenyl, evolves at 30.669 min. The spectrum of ferrous ions shows that the fourth crest is that of α-ethyl-α-methylbenzyl alcohol at 24.914 min. Finally, at 30.669 min, it displays the waveform of dicumyl peroxide (DCP).

According to individual normal curves obtained from DSC shown in Figure 2, after the addition of CuBr_2_ and FeBr_2_ to CHP, the heat wave peak appears twice. However, in the GC/MS experiments, within the first three substances, only CA repeated, as shown in each atlas. Furthermore, the derivatives CA and DCP were obtained by adding Cu^2+^ and Fe^2+^ in the CHP preparation process. It is predicted that this addition should generate metal ions that catalyze the formation of the two derivatives. Moreover, in the DSC experiment, the heating process in an exothermic reaction corresponds to the second thermal peak.

A comparison of the hazard posed by the mixture of CHP and incompatible metals in TAM III and reactions involving CHP contaminated with impurities show that the greatest hazard is posed by CHP mixed with Fe^2+^. The proposed mechanisms for metal-catalyzed decomposition [9] are as follows:

Fe^2+^ + C_6_H_5_C(CH_3_)_2_OOH → C_6_H_5_C(CH_3_)_2_O‧ + OH^−^ + Fe^3+^;
C_6_H_5_C(CH_3_)_2_OO‧ + C_6_H_5_C(CH_3_)_2_H → C_6_H_5_C(CH_3_)_2_OOH + C_6_H_5_C(CH_3_)_2_;
C_6_H_5_C(CH_3_)_2_OOH + H^+^ → [C_6_H_5_C(CH_3_)_2_O]^+^ + H_2_O;
[C_6_H_5_C(CH_3_)_2_O]^+^ → [C_6_H_5_O(CH_3_)_2_C]^+^;
C_6_H_5_C(CH_3_)_2_OOH + [C_6_H_5_O(CH_3_)_2_C]^+^ → [C_6_H_5_C(CH_3_)_2_O]^+^ + (CH_3_)_2_CO + C_6_H_5_OH;
C_6_H_5_C(CH_3_)_2_OOH → C_6_H_5_C(CH_3_)_2_O‧ + OH‧;
C_6_H_5_C(CH_3_)_2_O‧ + C_6_H_5_C(CH_3_)_2_H → C_6_H_5_C(CH_3_)_2_‧ + C_6_H_5_C(CH_3_)_2_OH;
C_6_H_5_C(CH_3_)_2_‧ + OH‧ → C_6_H_5_CH_3_C = CH_2_ + H_2_O

## 3. Materials and Methods

### 3.1. Samples

CHP, CuBr_2_, and ZnBr_2_, each with a concentration of 80.0 mass%, were provided by Alfa Aesar (Haverhill, MA, USA), and FeBr_2_ was purchased from Acros Organics (Taipei, Taiwan). Thermal hazard studies were conducted with 5.0 mass% CuBr_2_, ZnBr_2_, and FeBr_2_.

### 3.2. Differential Scanning Calorimetry

For determining the material energy, the sample and reference material were placed in different experimental containers. However, they were in the same heating furnace and were heated at a constant rate to measure enthalpy changes. The results were continuously recorded as an energy difference function. Dynamic scanning experiments were performed on a Mettler TA8000 system coupled with a DSC 821^e^ measuring test crucible (Mettler ME-26732) (Mettler-Toledo International Inc., Columbus, OH, USA). The test cell was sealed manually by using a kit equipped with Mettler’s differential scanning calorimetry [16,17]. The scanning rate selected for the temperature-programmed ramp was 4.0 °C/min, and the range of temperature rise was from 30.0 to 300.0 °C for tests.

### 3.3. Thermal Activity Monitor

The thermal activity monitor (TAM III) and its peripherals were manufactured by Thermometric, Inc., (Jarfalla, Sweden), and the operating temperature range was from 15.0 to 150.0 °C. The sensitivity level can reach 10.0 nW, and the temperature error is ±0.01 °C. TAM III [18,19,20] performs measurements in a cylinder, which is located in a 25.0-L constant temperature oil tank to maintain a highly constant temperature environment. There are two measuring cups (one for the sample and the other for the reference products) in each measurement cylinder. Every measuring cup was equipped with Peltier thermopile thermal sensors for measuring the thermal power released by the sample or reference products. The sample and reference products with Peltier detectors were connected in series. The signal principle is based on the sum of voltage signals from the paired detectors. Under isothermal conditions were detected 80.0, 90.0, 100.0 and 110.0 °C, we continuously measured the heat generated by substances during decomposition (gradual change in physical properties/resistance), and we titrated the incompatible chemical to measure its thermal stability. Five mass% ZnBr_2_, CuBr_2_, FeBr_2_ was added to 80.0 mass% CHP in TAM III thermostat tests at 80.0, 90, 100, and 110.0 °C, respectively.

### 3.4. Gas Chromatography and Gas Chromatography/Mass Spectrometer (GC/MS)

Gas chromatography (GC) [21] and gas chromatography/mass spectrometer were used to analyze a chemical mixture of interest. In GC, the sample is first heated and vaporized by the carrier gas. The vapors of the first sample can then be divided into individual components through a separation process, and volatile compounds contained in the sample can be identified from the peaks in the chromatogram. GC can be used for quantitative analysis as well as for determining the amount of various components. It is estimated that approximately 10%–20% of the materials can be analyzed using GC, but the material must be volatile and thermally stable. The temperature is set to be 5.0–10.0 °C which is greater than the boiling point for achieving optimal separation.

A mass spectrometer [22,23] is an analytical spectroscopic instrument that is primarily concerned with the separation of molecular (and atomic) species according to their mass. The MS does not actually measure the molecular mass directly, but rather the mass-to-charge ratio of the ions formed from the molecules.

With its heating mode with GC/MS, the products of qualitative analysis will use the product of each fragment peaks and GC/MS built-in database for comparison and analysis of possible species.

Test conditions:
  Column: Model no: Agilent 19091S–433, −60.0 °C–325.0 °C (350.0 °C), HP −5.0 ms, Capillary: 30.0 m × 250.0 μm × 0.25 μm.
  Carry gas: Helium (1.0 mL/min).
  Injection port: Split ratio (1:1), 280.0 °C, Injection volume: 1.0 μL.
  Detector: MSD, 280.0 °C.

## 4. Conclusions

Organic peroxides have substantial applications. In particular, they are widely used in industrial processes because of their high reactivity. In the DSC experiment, the parameter *T*_0_ was obtained as 105.0 °C, and thermal decomposition ∆*H*_d_ was 1,086.0 J/g. The addition of metal ions did not lead to any appreciable changes in ∆*H*_d_, but *T*_0_ increased considerably by 10.0–20.0 °C.

In the TAM III thermostat experiments, metal ions did not affect the CHP exothermic reaction. The value of *TMR*_iso_ of CHP mixed with Fe^2+^ at 80.0, 90.0, 100.0 and, 110.0 °C will appear during 0.34–0.43 h. TAM III test revealed that Fe^2+^ had the greatest influence on the extent of the CHP reaction. Iron is a major constituent of components used in manufacturing equipment. Therefore, the risk of a thermal disaster also increased substantially. The CHP manufacturing process should be performed very carefully.

GC/MS pyrolysis product analysis and CHP analysis indicated the presence of six significant peaks. Among them, the three largest peaks corresponded to cumene, AMS, and AP, which are common products in the CHP manufacturing process. In addition, they also appeared in other patterns, and they were not influenced by metal ions.

Investigating the GC/MS pattern of CHP containing added metal ions, we found the presence of anisole, CA, and DCP in the Zn^2+^, Cu^2+^, and Fe^2+^ patterns. Among them, CA and DCP posed a potential thermal hazard. We can predict the cause of the second peak.

During the manufacturing process, metal release may cause incompatible reactions and cause reactions to occur earlier, resulting in unforeseen events. If the monitoring system does not respond in a timely manner or adequately, thermal disasters are inevitable. Therefore, we should increase the inspection frequency of these equipment and components and periodically replace them to avoid incompatibility reactions, which can lead to disasters. If a component is corroded, it poses the risk of major accidents. Follow-up studies are required to find ways to prevent corrosions.

## Figures and Tables

**Table 1:** List of relevant chemical disasters worldwide since 2001.

Date	Country	Material	Cause	Hazard	Injuries	Deaths
06/09/2001	Australia	Cumene	Human error	Release	0	0
09/09/2002	USA	CHP	System failure	Explosion (Reactor)	0	0
09/24/2002	USA	CHP	Mechanical failure	Explosion	0	0
01/05/2003	USA	Cumene	Human error	Release	0	0
09/26/2003	Taiwan	CHP/DCPO	Thermal decomposition	Explosion (Reactor)	2	0
05/18/2005	Taiwan	Cumene	Accident (capsized)	–	0	0
05/24/2006	Taiwan	Phenol	Human error (splashed)	–	0	1
03/01/2007	USA	Cumene	Barge accident (leaked)	–	0	0
11/09/2007	Taiwan	Acetone	Fire (leaked)	Explosion (Tank)	0	0
01/30/2008	Taiwan	DCPO	Thermal decomposition	Explosion (Reactor)	0	0
01/08/2010	Taiwan	CHP	Fire	Explosion (Reactor)	0	0
07/19/2013	Taiwan	Phenol	Accident (capsized)	Release	1	0
04/04/2014	USA	Acetone	Thermal accident	Fire	0	0

Abbreviations used in the table: cumene hydroperoxide (CHP), dicumyl peroxide (DCPO).

**Table 2:** Nonisothermal data obtained from differential scanning calorimetry (DSC) tests for 80.0 mass% cumene hydroperoxide (CHP) mixed with ZnBr_2_, CuBr_2_, and FeBr_2_ at 4.0 °C.

Organic Peroxide		Incompatibility		*T*_0_ (°C)	*T*_max_ (°C)	Δ*H*_d_ (J/g)
	Mass (mg)	Substance	Mass (mg)			
CHP 80.0 mass%	2.5% ± 10%	–	–	105.0	156.0	1086.0
		ZnBr_2_	0.75	95.0	155.0	1192.0
		CuBr_2_	0.65	75.0	158.0	1100.0
		FeBr_2_	0.71	74.0	163.0	1246.0

**Table 3:** Heat flow for 80.0 mass% CHP mixed with ZnBr_2_, CuBr_2_, and FeBr_2_, tested using thermal activity monitor III ( TAM III ) scanning analysis at 80.0, 90.0, 100.0, and 110.0 °C.

Organic Peroxide	Incompatibility	80.0 °C		90.0 °C		100.0 °C		110.0 °C	
		*Q*_max_ (W/g)	*TMR*_iso_ (h)	*Q*_max_ (W/g)	*TMR*_iso_ (h)	*Q*_max_ (W/g)	*TMR*_iso_ (h)	*Q*_max_ (W/g)	*TMR*_iso_ (h)
CHP 80 mass%	–	–	–	0.0175	57.41	0.0293	22.88	0.0523	9.49
	ZnBr_2_	–	–	0.0050	29.42	0.0302	0.42	0.1011	0.41
	CuBr_2_	0.0105	23.35	0.0152	27.42	0.0307	8.66	0.1152	3.42
	FeBr_2_	0.0458	0.43	0.1005	0.35	0.0771	0.34	0.0932	0.37

**Table 4:** Apparent activation energy of CHP decomposition after mixing CHP with ZnBr_2_, CuBr_2_, or FeBr_2_ individually; the apparent activation energy was obtained using the Arrhenius equation.

Material Contaminant		*E_a_* (kJ/mol)
CHP	–	155.46
CHP	ZnBr_2_	146.76
CHP	CuBr_2_	95.55
CHP	FeBr_2_	21.81

**Table 5:** Apparent activation energy values of CHP obtained from the literature.

Authors	*E_a_* (kJ/mol)
Somma, *et al.* [6]	139.4 ± 0.8
Huang, *et al.* [10]	161.96
Chen, *et al.* [5]	180.176
Yuan Lu, *et al.* [11]	138.7 ± 5.4
Duh, *et al.* [7]	122.0, 125.0
